# Epidermal growth factor induces platelet-activating factor production through receptors transactivation and cytosolic phospholipase A_2_ in ovarian cancer cells

**DOI:** 10.1186/1757-2215-7-39

**Published:** 2014-04-11

**Authors:** Yi Yu, Xiaoyan Zhang, Shanshan Hong, Mingxing Zhang, Qingqing Cai, Wei Jiang, Congjian Xu

**Affiliations:** 1 Obstetrics and Gynecology Hospital, Fudan University, No.419 Fang-Xie Road, Shanghai 200011, People’s Republic of China; 2 Department of Obstetrics and Gynecology of Shanghai Medical School, Fudan University, No.138 Yi-Xueyuan Road, Shanghai 200032, People’s Republic of China; 3 Shanghai Key Laboratory of Female Reproductive Endocrine Related Diseases, No. 413 Zhao-Jiabang Road, Shanghai 200011, People’s Republic of China; 4 Institute of Biomedical Sciences, Fudan University, No.138 Yi-Xueyuan Road, Shanghai 200032, People’s Republic of China

**Keywords:** Ovarian cancer, EGF, EGF-receptor, PAF-receptor, ERK, cPLA_2_

## Abstract

**Background:**

Among the pro-inflammatory lipid mediators, platelet-activating factor (PAF) is a major primary and secondary messenger that binds to the PAF-receptor (PAFR). Epidermal growth factor (EGF) is a polypeptide growth factor that binds to the EGF-receptor (EGFR). Evidence suggests that both PAF and EGF play a significant role in oncogenic transformation, tumor growth, neoangiogenesis and metastasis, including ovarian cancer. PAF has the potential to transactivate EGFR in ovarian cancer cells. This study explores the mechanisms involved in EGF-induced PAF production.

**Methods:**

The effect of EGF on PAF production in ovarian cancer cells was observed using enzyme-linked immunosorbent assay. The receptors transactivation and the role of cytosolic phospholipase A_2_ (cPLA_2_) in modulating PAF production induced by EGF was assessed using pharmacological inhibitors, si-RNA knockdown, targeted gene overexpression and immunocytochemistry. The signaling pathways invovled in PAF production induced by EGF in ovarian cancer cells were assessed.

**Results:**

We demonstrate that EGF increases the production of PAF in CAOV3 and SKOV3 ovarian cancer cell lines. EGF induces the transactivation of PAFR, which can be blocked by an EGFR inhibitor. Inhibition of EGFR and/or PAFR blocks PAF production in response to EGF. EGF-induced PAF production involves the phosphorylation of extracellular-regulated protein kinase (ERK) and cytosolic phospholipase A_2_ (cPLA_2_). A cPLA_2_ inhibitor blocks EGF-induced PAF production as well as si-cPLA_2_, while overexpression of cPLA_2_ increases PAF production.

**Conclusions:**

These results indicate that EGF stimulates PAF production in ovarian cancer cells in a manner that requires cPLA_2_. We have also determined that crosstalk can occur bidirectionally between EGFR and PAFR, suggesting that EGF-induced PAF production could result in positive feedback that acts on the PAF-receptor to promote ovarian cancer progression.

## Background

Chronic inflammatory microenvironments have been suggested as the major predisposing factor for ovarian and other cancers [1]. Lipid mediators such as lysophosphatidic acid (LPA) and prostaglandins (PGs), with their specific receptors and pathways, have been shown to play a critical role in cancer initiation and progression [2-4]. Platelet activating factor (PAF, 1-O-alkyl-2-acetyl-sn-glycero-3-phosphorylcholine) is also one of the most potent lipid mediators involved in many different biological pathways in inflammatory diseases and cancers [5,6]. There are two distinct pathways in which PAF can be synthesized: the de novo pathway and the remodeling pathway [7]. The de novo pathway is used to maintain PAF levels during normal cellular function, while the remodeling pathway is activated by inflammatory agents and is the primary source of PAF under pathological conditions. The initiation of the remodeling pathway requires membrane phospholipid hydrolysis by phospholipase A_2_ (PLA_2_), which supplies lyso-PAF, a precursor of PAF. Lyso-PAF acetyltransferase then converts lyso-PAF into PAF and finally, PAF activates the PAF-receptor (PAFR), a member of the superfamily of G protein-coupled receptors [8,9]. These events are thought to play an important role in the oncogenic transformation [10], proliferation [11] and metastasis [12] of several types of cancers, including ovarian cancers. However, PAF is rapidly degraded by PAF acetylhydrolases (PAF-AH), which cleaves the acetyl group at the *sn*-2 position to reform back to lyso-PAF [13]. Therefore, there maybe a possibility that PAF acts as an autocrine growth factor to promote ovarian cancer progression.

PLA_2_ is classified into three groups: group VI calcium-independent PLA_2_s (iPLA_2_s), secretory PLA_2_s (sPLA_2_s), and group IV cytosolic PLA_2_s (cPLA_2_) [14]. Group IVA cPLA_2_ is essential for producing PAF because PAF synthesis is significantly diminished in calcium ionophore-stimulated macrophages derived from group IVA cPLA_2_-deficient mice compared with those from wild-type mice [15]. However, the role of cPLA_2_ in growth factor-mediated PAF production in ovarian cancer cells has not been examined. The current study focuses on the role of cPLA_2_ in epidermal growth factor (EGF)-stimulated PAF production in ovarian cancer cells. Epidermal growth factor (EGF), a polypeptide growth factor, binds to the EGF-receptor (EGFR), which is a transmembrane protein tyrosine kinase. EGF is free of sugar groups, has excellent chemical stability and is highly prevalent in human blood. It also has been reported that EGF stimulates proliferation of ovarian cancer cells and other types of carcinoma cells [16,17].

Our earlier study demonstrated that ovarian cancer cells express high levels of PAFR as well as that PAF can stimulate transactivation of the EGFR in ovarian cancer cells [18]. PAF can activate matrix metalloproteases, which cleave pro-EGF from the membrane to release active ligands. Intracellular signaling molecules, such as phospholipase C and protein kinase A, have also been suggested as mediators of PAF-induced transactivation of receptor tyrosine kinase. Conversely, some growth factors and cytokines can activate PAF production, resulting in transactivation of the PAF receptor [19]. However, it has not yet been tested whether reverse crosstalk occurs and whether EGF can stimulate activation of PAFR.

In this study, we used CAOV3 and SKOV3 adenocarcinoma cells, two well-characterized human ovarian cancer cell lines, as models to examine the mechanisms involved in EGF-induced PAF production. Stimulation of cells with EGF increases PAF levels in the medium. This response can be inhibited by either EGFR or PAFR inhibition. EGF recruits cPLA_2_ by activating the ERK signaling pathway and overexpression of cPLA_2_ increases PAF production, while inhibition of cPLA_2_ blocks EGF-induced PAF production. These results show that EGF activates cPLA_2_, that cPLA_2_ is involved in the production of PAF and that bidirectional crosstalk can occur between the EGF and the PAF receptors.

## Materials and methods

### Cell culture and chemical reagents

The ovarian cancer cell lines CAOV3 and SKOV3 (obtained from the Cell Bank of the Chinese Academy of Science, Shanghai, China) were maintained at 37°C in a humidified 5% CO_2_ atmosphere in RPMI-1640 medium with 10% fetal calf serum (Gibco, Invitrogen, Carlsbad, CA), 100 IU/ml penicillin G, and 100 mg/ml streptomycin sulfate (Sigma-Aldrich, St. Louis, MO). Cells were serum starved by incubation in serum-free medium for 12–24 hours before the start of the experiments. Lipofectamine 2000 Transfection Reagent and Opti-MEM-1 Medium (Invitrogen, Carlsbad, CA) were used for plasmid and siRNA transfection. The vector encoding cPLA_2_ and cPLA_2_-targeted siRNA were synthesized by Shanghai GenePharma Co. AG1478 (EGFR inhibitor) [20] and WEB2086 (PAFR inhibitor) were purchased from Sigma-Aldrich (St Louis, MO). PD98059 (ERK inhibitor) and LY294002 (PI3K inhibitor) were obtained from Cell Signaling Technology (Boston, MA). Rabbit polyclonal antibodies that were used in this study were directed against phospho/total-EGFR, phospho/total-PLCβ, phospho/total-cPLA_2_, phospho/total-Akt, and phospho/total-ERK. All the antibodies were purchased from Cell Signaling Technology Co. The mouse monoclonal antibodies that were used in this study were directed against actin (Sigma, Missouri, USA).

### Western blot analysis

Cellular extracts were prepared in modified radioimmunoprecipitation assay (RIPA) buffer (50 mM Tris–HCl pH 7.4, 1% NP-40, 0.25% Na-deoxycholate, 150 mM NaCl, 1 mM EDTA, 1 mM PMSF, protease inhibitor cocktail). Protein concentrations of cellular extracts were measured using a Bio-Rad protein assay kit. Then, cellular extracts were subjected to SDS-PAGE. Proteins were transferred to PVDF membranes. After blocking for 1 h at room temperature in 5% BSA, blots were probed with the primary antibody at a 1:1000 dilution and incubated overnight at 4°C. Subsequently, blots were washed three times and incubated for 1 h at room temperature with a 1:10000 dilution of secondary peroxidase-conjugated antibodies. Following three washes, immunoreactive bands were detected using electrochemiluminescence (ECL).

### Transfection with cPLA_2_ overexpression vector

Cells were seeded and grown to approximately 40% confluence. Cells were then transfected with the CMV-MCS-EGFP-SV40-Neomycin-cPLA2 expression vector using Lipofectamine2000. Briefly, cells were transfected for 48 hours with 1 μg of DNA and were then serum-starved for 12 hours before experimentation.

### Incubation with small interference RNA for cPLA_2_

Cells were seeded and grown to approximately 40% confluence. Cells were incubated with 50 nM siRNA for cPLA2 or non-target siRNA, using Lipofectamine 2000 for 48 hours, based on the manufacture’s protocol. Cells were then serum starved for 12 hours before experimentation.

### PAF assay

Aliquots of the supernatant from stimulated ovarian cancer cells were collected and the PAF concentrations were measured using a specific enzyme immunoassay kit (Cayman Chemical, Ann Arbor, MI). The assay was performed according to the manufacturer’s instructions. PAF production was evaluated in duplicates, and the concentrations were determined from a standard curve of PAF. The sensitivity of the assay allowed for the detection of up to 15 pg/ml. When necessary, the samples were diluted in the assay buffer.

### Immunocytochemistry

After the drug treatment, the cells were fixed with 100% methanol for 6 min at −20°C, then washed with PBS and left at 4°C until use. Cells were permeabilized by incubation in PBS containing 0.3% Triton X-100 and 5% goat serum for 30 min. A polyclonal antibody against phospho-cPLA_2_ was used at a 1:100 dilution, and a secondary antibody FITC-conjugated goat anti-rabbit (Invitrogen, Carlsbad, CA), was used at a 1:200 dilution. The first antibody was incubated overnight at 4°C and the second antibody for 2 hours at RT. Images were captured with an Olympus DP 71 camera (Tokyo, Japan). The magnification level was 400 ×.

### Statistical analysis

All experiments were performed at least three times. The data are expressed as the mean ± SD. Wherever appropriate, the data were also subjected to unpaired, two-tailed Student’s t-tests. Differences were considered significant when *P* < 0.05.

## Results

### Effects of EGF on PAF production in ovarian cancer cells

**Figure 1:**
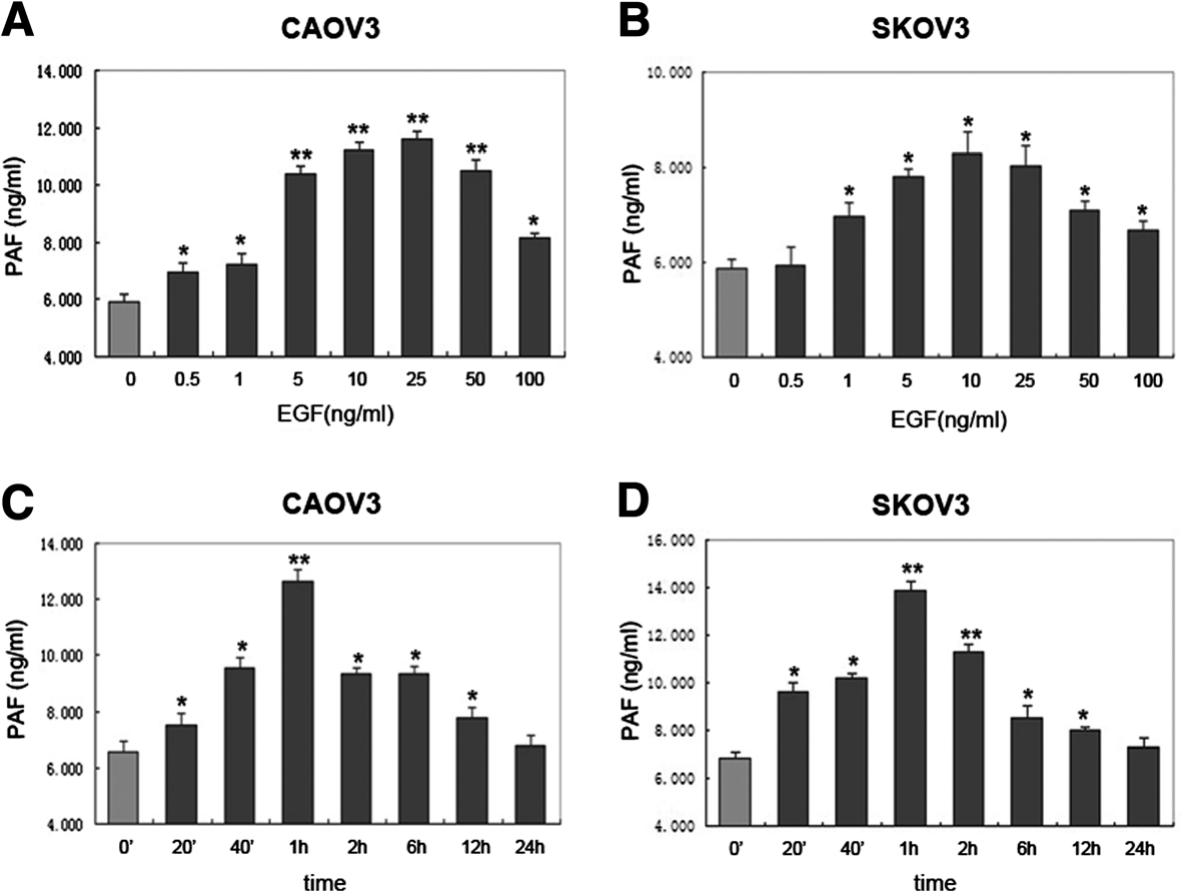
**Effects of epidermal growth factor (EGF) on platelet-activating factor (PAF) production in ovarian cancer cells. (A and B)** Release of PAF in response to increasing concentrations of EGF for 1 h. CAOV3 and SKOV3 cells were serum starved and then stimulated with indicated concentrations (0.5 to 100 ng/ml) of EGF. **(C and D)** Time course of PAF increase in response to 10 ng/ml EGF. CAOV3 and SKOV3 cells were treated with EGF for the indicated time (20 min to 24 h) while measuring PAF production. Bars represent the average of triplicates ± S.D.; “*” (p < 0.05) and “**” (p < 0.01) indicate a statistically significant difference compared to the untreated control.

As shown in Figure 1, in CAOV3 ovarian cancer cell lines, extracellular EGF caused a significant rise in the PAF released from 0.5 ng/ml to 100 ng/ml; while in SKOV3 ovarian cancer cell lines, ext racellular EGF caused a significant rise in the PAF release from 1 ng/ml to 100 ng/ml. The maximum effect was reached with 25 ng/ml of EGF (1.952 ± 0.9-fold) in CAOV3 (Figure 1A) cells and 10 ng/ml EGF (1.414 ± 0.3-fold) in SKOV3 cells (Figure 1B). Both in CAOV3 and SKOV3 cells, PAF production increased after 20 min of stimulation with EGF (10 ng/ml) and continued to rise to a maximum after 1 h. Longer stimulation of EGF (24 h) caused no significant additional increase in PAF release over that obtained at 1 h (Figure 1C and D). Together, our data indicated that EGF stimulates PAF production in two human ovarian cancer cell lines.

### EGFR transactivates PAFR followed by EGF stimulation

**Figure 2:**
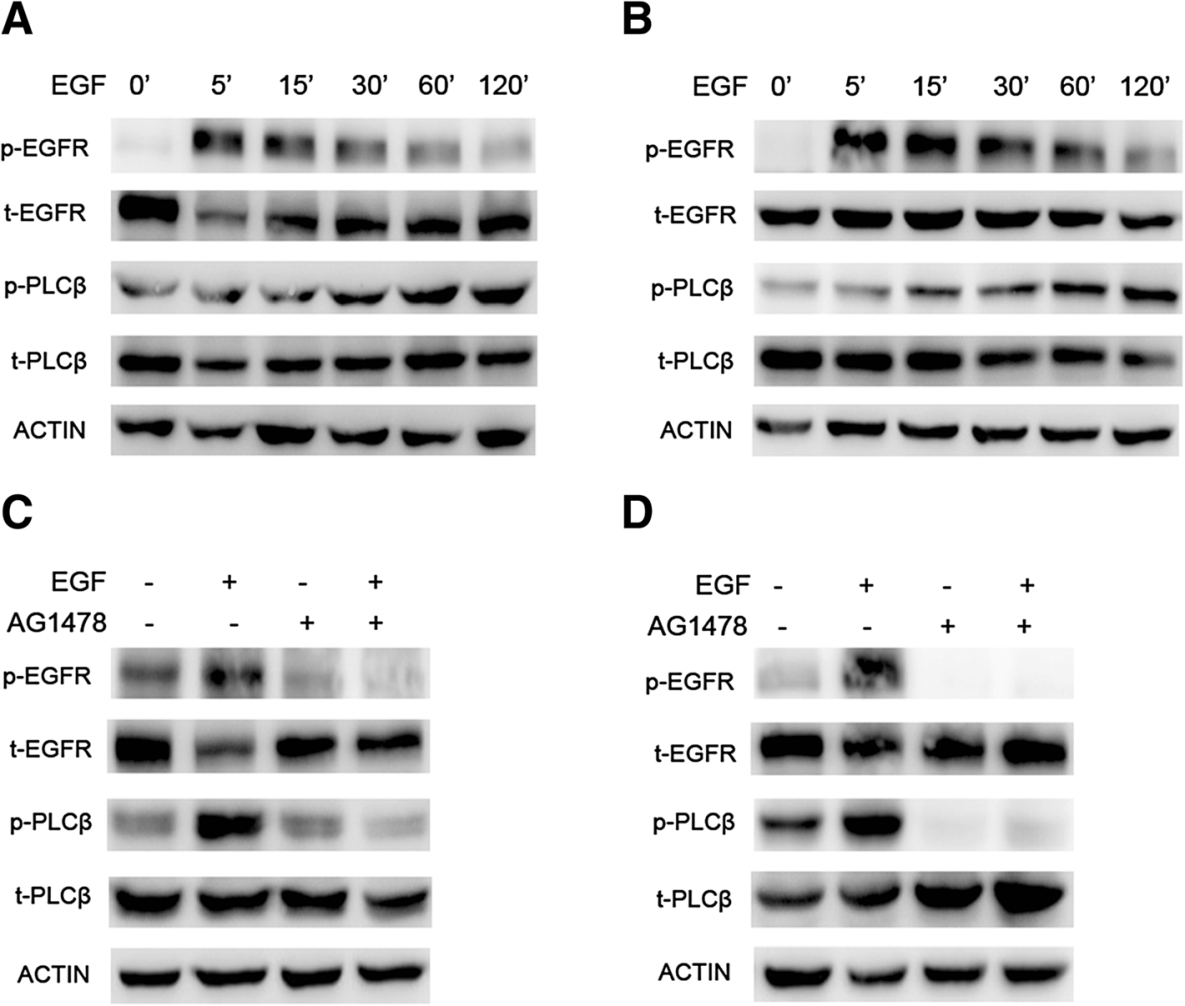
**PAFR-coupled PLCβ phosphorylation induced by EGF in ovarian cancer cells.** CAOV3 **(A)** and SKOV3 **(B)** cells were treated with a constant dose (10 ng/ml) of EGF for different time intervals, as indicated. Total protein was extracted and analyzed for phospho-EGFR/total-EGFR and phospho-PLCβ/total-PLCβ and was examined by immunoblot analysis. β-actin was used as an internal control. CAOV3 **(C)** and SKOV3 **(D)** cells were pretreated with 10 μM AG1478 for 1 h before exposure to 10 ng/ml EGF for 10 min. Total protein was extracted and analyzed for phospho-EGFR/total-EGFR and phospho-PLCβ/total-PLCβ and was examined by immunoblot analysis. β-actin was used as the internal control. Bars represent the average of the triplicates ± S.D.; “*” (p < 0.05) and “**” (p < 0.01) indicate a statistically significant difference compared to the untreated control.

Our previous data has shown that PAF can transactivate the EGFR, and EGF and PAF are shown to activate many of the same intracellular signaling pathways. Conversely, to test whether a growth factor might transactivate PAFR, we first stimulated CAOV3 and SKOV3 cells with 10 ng/ml of EGF for varying times (5 min to 120 min) to observe the change in phosphorylation of EGFR and phosphoinositide-specific phospholipase C-β (PLCβ). PLCβ has been shown to lie downstream of the activated PAFR and, therefore, the phosphorylation of PLCβ indicates the PAFR activation [21-23]. As shown in Figure 2A and B, stimulation with EGF (10 ng/ml) evoked EGFR and PLCβ phosphorylation in a time-dependent manner in CAOV3 and SKOV3 cells. Phosphorylation of EGFR reached maximum activation at 5 min, followed by a subsequent reduction to the baseline by 120 min. Meanwhile, the phosphorylation of PLCβ increased gradually and reached a maximum activation at 120 min. We also observed that in both the cells, the phosphorylation of PLCβ was somewhat slower than the phosphorylation of EGFR after stimulation with EGF, suggesting the mechanism of EGF-induced transactivation of PAFR.

We next investigated whether EGF-induced PAFR transactivation is EGFR-dependent. Serum-starved CAOV3 and SKOV3 cells were treated with AG1478 (10 μM), an EGFR-specific tyrosine kinase inhibitor, for 1 h before exposing them to EGF (10 ng/ml) for 10 min. As shown in Figure 2C and D, AG1478 inhibited the phosphorylation of EGFR and PLCβ, in both cells, with or without EGF stimulation. These results suggest that EGF can induce PAFR transactivation and that PAFR transactivation is EGFR-dependent.

### Effects of EGFR and PAFR inhibition on PAF production in ovarian cancer cells

**Figure 3:**
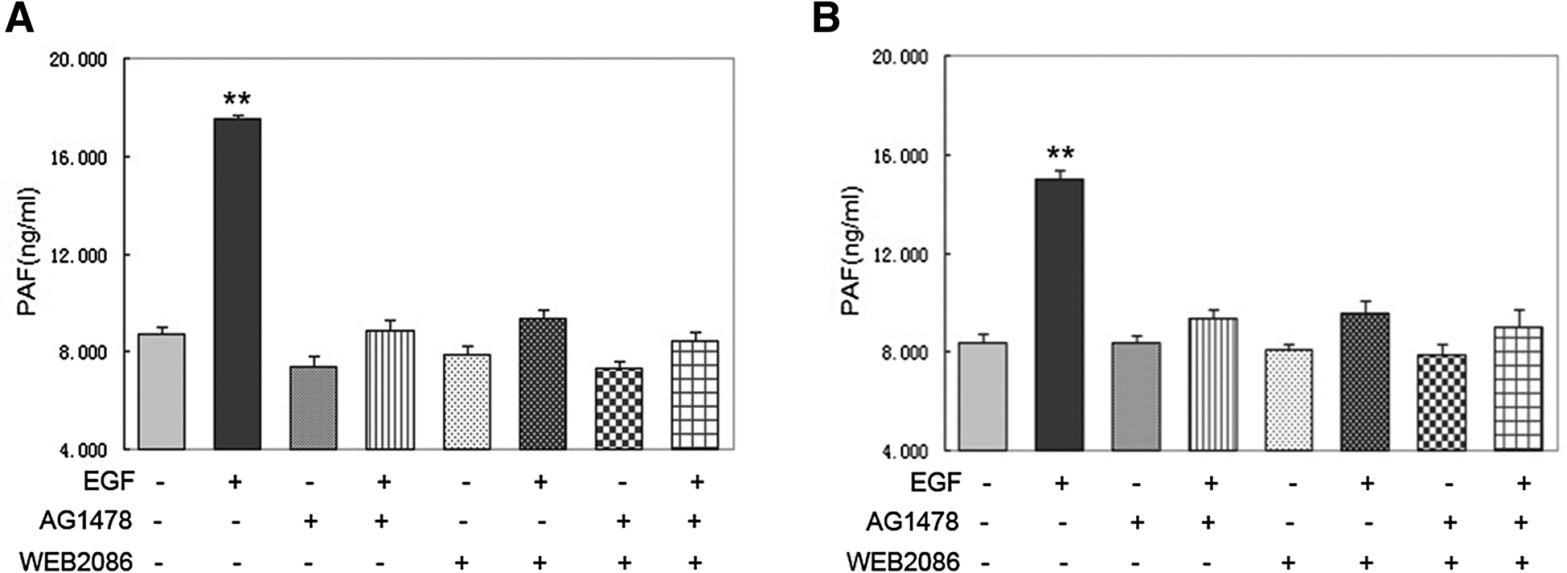
**Effects of an EGFR inhibitor and PAFR inhibitor on EGF-induced PAF production.** CAOV3 **(A)** and SKOV3 **(B)** cells were serum starved and then pretreated with 10 μM AG1478 or 50 μM WEB2086 or a combination of AG1478 and WEB2086 for 30 min. Cells were then stimulated with 10 ng/ml EGF for 30 min. Medium was harvested, and the amount of PAF was measured. In **A** and **B**, bars represent the average of triplicates ± S.D.; “*” (p < 0.05) and “**” (p < 0.01) indicate a statistically significant difference compared to the untreated control.

EGF-induced PAF production is presumably mediated through EGFR-mediated activation of phospholipases. To address the potential for crosstalk between EGFR and PAFR, we tested whether EGFR and PAFR activation correlated with increased PAF production. AG1478 (an EGFR-specific tyrosine kinase inhibitor) and WEB2086 (a small molecular inhibitor of PAFR) were used to block EGFR and PAFR activation. CAOV3 and SKOV3 cells were pretreated with 10 μM of AG1478 or 50 μM of WEB2086 or a combination of AG1478 and WEB2086 for 30 min. The cells were then incubated with 10 ng/ml of EGF for 30 min, and PAF production was measured using an ELISA assay. As shown in Figure 3, AG1478 and WEB2086 significantly reduced the EGF-induced increase in PAF levels in both ovarian cancer cell lines. Additive PAF-production-inhibiting effects were observed on the combined inhibition of both receptors. Taken together, these results suggest that both EGFR and PAFR are involved in EGF-induced PAF production.

### Akt and ERK lie downstream of activated EGFR and PAFR, and ERK is required for activation of cPLA_2_

**Figure 4:**
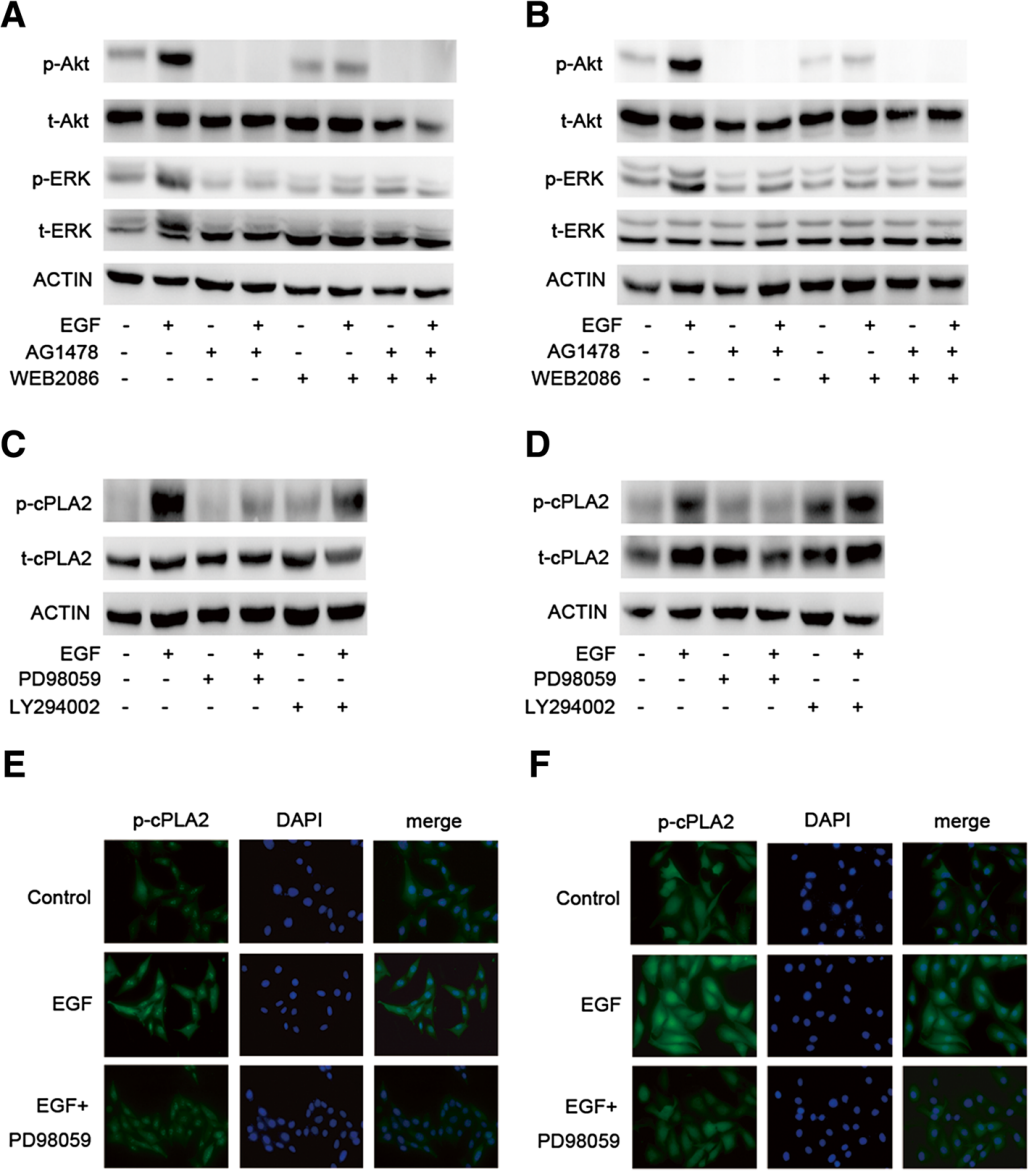
**Akt and ERK phosphorylation induced by EGF in ovarian cancer cells and ERK is required for activation of cPLA**_**2**_**.** CAOV3 **(A)** and SKOV3 **(B)** cells were pretreated with AG1478 (10 μM) and/or WEB2086 (50 μM) for 1 h before exposure to EGF (10 ng/ml) for 10 min. Total protein was extracted and analyzed for phospho-Akt/total-Akt and phospho-ERK/total-ERK and was examined by immunoblot analysis. β-actin was used as a loading control. CAOV3 **(C)** and SKOV3 **(D)** cells were pretreated with the ERK inhibitor PD98059 (10 μM) and the Akt inhibitor LY294002 (10 μM) before exposure to EGF (10 ng/ml) for 10 min. Total protein was extracted and analyzed, and phospho-cPLA_2_/total-cPLA_2_ was examined by immunoblot analysis. β-actin was used as a loading control. For the immunofluorescence staining of phosphorylated cPLA_2_ in CAOV3 **(E)** and SKOV3 **(F)** cells, after 10 min of incubation without any drug or with 10 ng/ml of EGF or with 10 ng/ml of EGF plus 10 μM of PD98059, cells were labeled with polyclonal antibody to phosphorylated cPLA_2_ overnight, and then cells were incubated with fluoresent secondary antibody to phospho- cPLA_2_ for 1 h and stained with DAPI for 10 min.

We next investigated the signaling pathway downstream of activated EGFR and PAFR in ovarian cancer cells to elucidate the mechanisms involved in EGF-induced PAF production. Western blots using an antibody that specifically recognized the phosphorylated forms of Akt and ERK were used. As shown in Figure 4A and B, exposure to 10 ng/ml of EGF caused the rapid phosphorylation of Akt and ERK in CAOV3 and SKOV3 cells. We then investigated the effects of the EGFR inhibitor, AG1478, and the PAFR inhibitor, WEB2086, to determine whether both EGFR and PAFR were involved in Akt and ERK activation using EGF. Preincubation with 10 μM of AG1478 and/or 50 μM of WEB2086 for 1 h totally prevented the activation of Akt and ERK, following stimulation using 10 ng/ml of EGF for 10 min. These data suggest that EGF activates downstream Akt and ERK signaling via EGFR and PAFR.

To further analyze the mechanisms of EGF-induced PAF production, we examined what is required for activation of cPLA_2_ among the activated downstream signaling pathways. As shown in Figure 4C and D, exposure to EGF caused the significant phosphorylation of cPLA_2_ in CAOV3 and SKOV3 cells. The activation of cPLA_2_ was treated using 10 ng/ml of EGF for 10 min. The phosphorylation of cPLA_2_, stimulated by EGF, could be blocked using by 10 μM of PD98059 (an ERK inhibitor), while no inhibition was observed with LY294002 (10 μM), which binds to the site of PI3K, thereby preventing the activation of target enzymes, such as Akt. Furthermore, the staining intensity of phosphorylated of cPLA_2_, following 10 min of 10 ng/ml EGF treatment, was much higher in CAOV3 and SKOV3 cells than in control cells, and when cells were pretreated with the ERK inhibitor PD98059, the staining intensity of phosphorylated of cPLA_2_ was reduced (Figure 4E and F). These data indicate that ERK is required for the phosphorylation of cPLA_2_ in ovarian cancer cells.

### Effects of cPLA2 on EGF-induced PAF production

**Figure 5:**
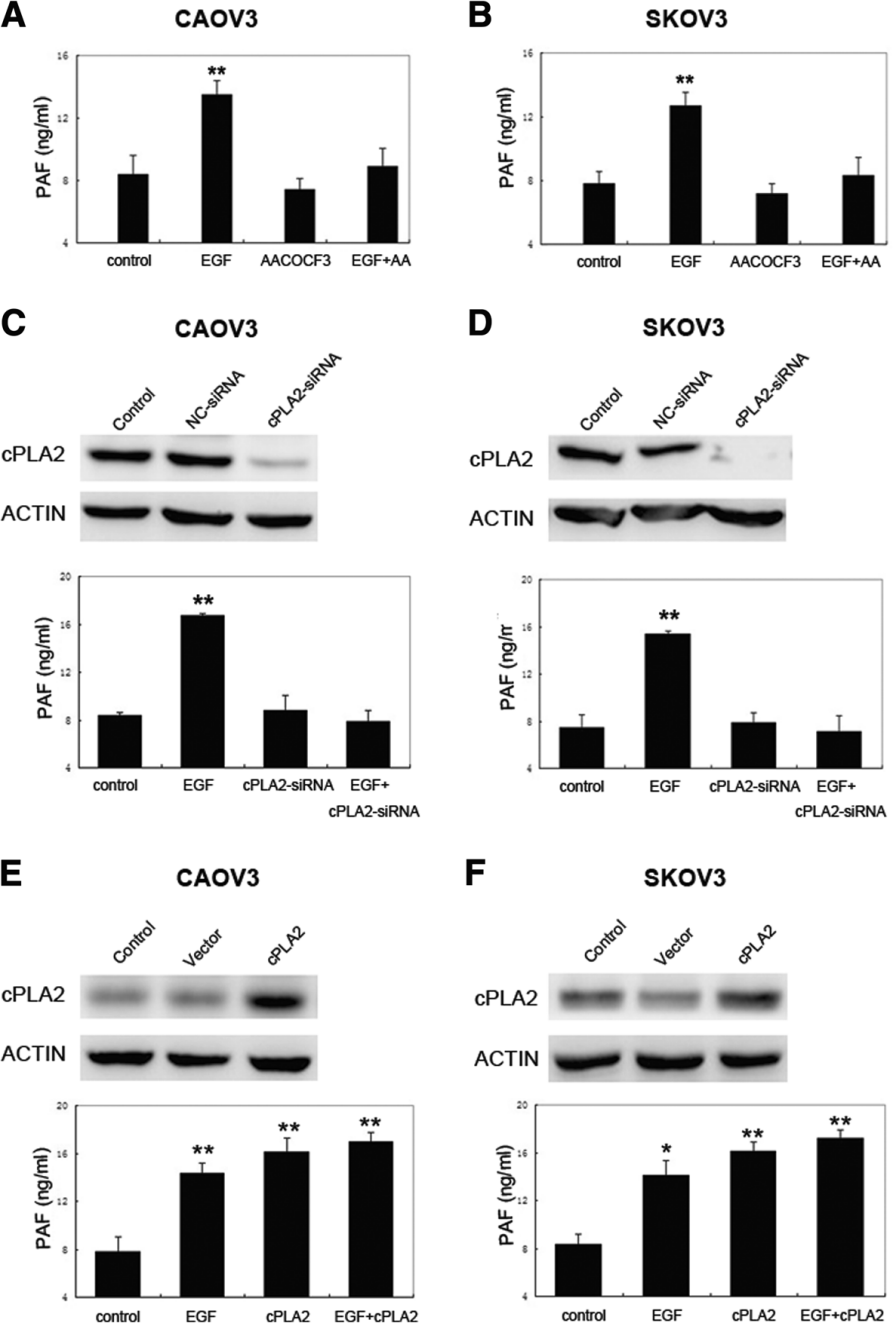
**Role of cytosolic phospholipase A**_**2 **_**(cPLA**_**2**_**) in EGF-induced PAF production in ovarian cancer cells.** CAOV3 **(A)** and SKOV3 **(B)** cells were serum starved and then pretreated with the cPLA_2_ inhibitor AACOCF3 (10 μM) for 30 min. Cells were then stimulated with 10 ng/ml EGF for 30 min. Medium was harvested, and the amount of PAF was measured. **(C and D)** CAOV3 and SKOV3 cells were transfected with a negative-control (NC) or cPLA_2_-targeted siRNAs. Following 48 h of transfection, cells were treated with EGF (10 ng/ml) for 30 min. Medium was harvested, and the amount of PAF was measured. **(E and F)** CAOV3 and SKOV3 cells were transfected with the cPLA_2_ overexpression vector for 48 h. Cells were then treated with EGF (10 ng/ml) for 30 min. Medium was harvested, and the amount of PAF was measured. Bars represent the average of triplicates ± S.D.; “*” (p < 0.05) and “**” (p < 0.01) indicate a statistically significant difference compared to the untreated control.

To determine whether cPLA_2_ is essential for producing PAF stimulated by EGF in ovarian cancer cells, we first tested for the effects of arachidonyl trifluoromethyl ketone (AACOCF3), a small-molecular inhibitor of cPLA_2_[24,25] on PAF production. As shown in Figure 5A and B, CAOV3 and SKOV3 cells were pretreated with AACOCF3 (10 μM) for 30 min before exposing the cells to EGF (10 ng/ml) for 30 min. cPLA_2_ inhibition by AACOCF3 restained PAF production induced by EGF in both cells. To further analyze the effect of cPLA_2_ inhibition on PAF production, we suppressed cPLA_2_, through RNA interference in CAOV3 and SKOV3 cells before the stimulation with EGF. Our data suggest that cPLA_2_-targeted siRNA was able to effectively silence the expression and that the suppression of cPLA_2_ led to the inhibition of PAF production when cells were exposed to EGF compared to EGF alone (Figure 5C and D). Furthermore, we overexpressed cPLA_2_ in CAOV3 and SKOV3 cells using an expression vector encoding cPLA_2_ to determine whether cPLA_2_ is involved with EGF-induced PAF production. As shown in Figure 5E and F, the vector encoding cPLA_2_ significantly increased the expression in CAOV3 and SKOV3 cells, and the overexpression of cPLA_2_ enhanced the PAF production over control cells. However, EGF did not further increase the PAF production in cells overexpressing cPLA_2,_ which may reflect a limit to the amount of PAF that can be produced. Together, our data suggest a role for cPLA_2_ in EGF-induced production in ovarian cancer cells.

## Discussion

**Figure 6:**
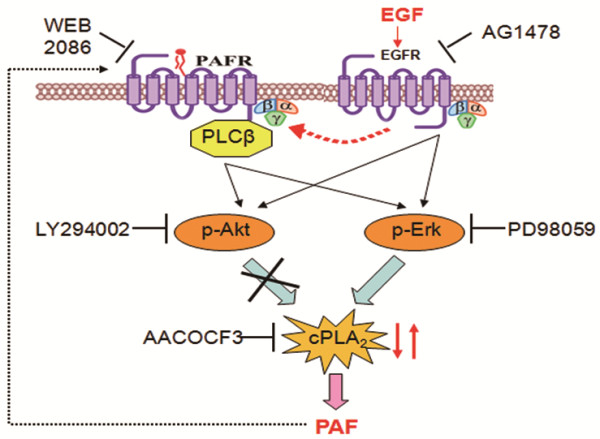
**Proposed cascade of the mechanisms involved in PAF production by extracellular EGF in ovarian cancer cells.** Extracellular EGF binds to the EGF-receptor (EGFR) and transactivates the PAFR. Activation of EGFR and PAFR stimulates the phosphorylation of Akt and ERK. Only the ERK pathway induces the phosphorylation of cPLA_2_, the latter increases the production of PAF.

The results of this study demonstrate that EGF stimulates the release of PAF from human ovarian cancer cells by acting on the EGF-receptor and transactivating the PAF-receptor. Stimulation of EGFR and PAFR led to the activation of Akt and ERK, but only the phosphorylation of ERK could stimulate the cPLA_2_ enzyme, resulting in the production of PAF (Figure 6).

This is the first study to examine the role of EGF, a mitogenic agonist that binds to EGFR, a tyrosine kinase receptor, on PAF production in ovarian cancer cells. Overexpression of EGFR is common in cancers, including 35-70% of ovarian cancers [26-28]. The release of PAF stimulated by EGF can be blocked by AG1478, an inhibitor of EGFR, as expected. Interestingly, WEB2086, an inhibitor of PAFR, also blocks EGF-stimulated PAF production, suggesting that crosstalk between the receptors (EGFR and PAFR) is required. We have previously observed that the tyrosine phospho-activation of protein targets, including EGFR, were significantly increased after PAF treatment, and that phosphorylation was blocked or inhibited by the PAFR antagonist Ginkgolide B using phospho-antibody microarray technologies [18]. Phosphoinositide-specific phospholipase C (PLC) plays a significant role in transmembrane signaling and the subfamily of PLCβ are activated by the G-proteins. Our previous data revealed that PAF can activate PLCβ-dependent PKC and Ca^2+^ pathways via PAFR to promote ovarian cancer progression. In this study, we demonstrate that EGF stimulates the phosphorylation of PLCβ, which can be blocked by the EGFR inhibitor AG1478, suggesting that the crosstalk occurs bidirectionally between EGFR and PAFR in ovarian cancer cell lines.

PAFR expression is elevated in non-mucinous types of ovarian cancer tissues and cells, suggesting its role in the pathogenesis and progression of ovarian cancer. PAF, the sole ligand of PAFR, is secreted by many different cell types, including endothelial, stromal and inflammatory cells, as well as many different tumor cells [29-31], thus indicating an important role of PAF/PAFR signaling in ovarian cancer progression. In addition, it has been showen that PAF/PAFR significantly promotes ovarian cancer proliferation and invasion [32,33]. The mechanisms, however, in which PAF accumulates in the extracellular space to activate PAFR is still unknown. PAF may be produced within the cell membrane and then exported out of the cell, or it may be synthetized extracellularly. In the present study, we observed that EGF treatment led to an increased production of PAF. As we have demonstrated that PAF-induced ovarian cancer cell proliferation and invasion is dependent on PAFR, it can be assumed that PAF is an autocrine growth factor for ovarian cancer.

The current study demonstrates that EGF stimulates the phosphorylation of Akt and ERK, which can be blocked by either AG1478, an inhibitor of EGFR, or WEB2086, an inhibitor of PAFR. This suggests that EGFR and PAFR, stimulated by EGF, can potentially activate common downstream intracellular signaling pathways. ERK inhibition with PD98059 completely abolishes the phosphorylation of cPLA_2_, while the antagonist of Akt had no effect on the activation of cPLA_2_, suggesting that the phosphorylation of cPLA_2_ induced by EGF is ERK-dependent. In rat articular chondrocytes, the phosphorylation of ERK and p38 MAPKs activated cPLA_2_ and increased PGE2 production, which is another type of lipid mediator, similar to PAF [34]. Phosphorylated ERK in dorsal root ganglion neurons, caused by spinal cord injury, can induce increased levels of PGE2 [35]. Whether EGF could affect the expression of cPLA_2_ and whether cPLA_2_ could affect the production of PAF requires further research in ovarian cancer cells.

cPLA_2_ can be activated by small GTPases, receptor tyrosine kinases, and phosphatidylinositides [36,37]. In this study, we have shown that the phosphorylation of cPLA_2_ is stimulated by EGF in ovarian cancer cells. Further, we have shown that cPLA_2_ is likely to be involved in PAF production, as both of the specific cPLA_2_ inhibitors, AACOCF3 and cPLA2-targeted siRNA, block PAF production, while exogenously added cPLA_2_ promotes PAF production. The role of cPLA_2_ in smooth muscle cell spreading and/or migration has also been well documented [38]. The results concerning the role of cPLA_2_ in EGF-induced PAF production, along with the convergence of signaling molecules on cPLA_2_, suggest that cPLA_2_ may be a potential therapeutic target in ovarian cancer.

## Conclusions

Taken together, our results identify mechanisms leading to PAF production and reveal a novel autocrine loop in ovarian cancer cells. Extracellular EGF could stimulate the release of PAF, and this signaling pathway depends on the transactivation between EGFR and PAFR. This requires the phosphorylation of ERK and cPLA_2_, though the activation of Akt is not involved in this pathway.

## Abbreviations

PAF: Platelet-activating factor; PAFR: PAF-receptor; EGF: Epidermal growth factor; EGFR: EGF-receptor; ERK: Extracellular-regulated protein kinase; cPLA2: cytosolic phospholipase A_2_; siRNA: Short interfering RNA; ELISA: Enzyme-linked immunosorbent assay; PLCβ: Phospholipase C-β; AG1478: An EGFR-specific tyrosine kinase inhibitor; WEB2086: A small molecular inhibitor of PAFR; AACOCF3: Arachidonyl trifluoromethyl ketone; PD98059: ERK inhibitor; LY294002: PI3K inhibitor.

## Competing interest

The authors indicated that they have no conflicts of interests with regard to the content of this paper.

## Authors’ contributions

YY performed the experiments and drafted the manuscript. XYZ and SSH participated in the design of this study. MXZ and QQC participated in the experiments. WJ and CJX contributed to the design of this study, final data analysis and edited the manuscript. All authors read and approved the final manuscript.
